# Flexing and downsizing the femoral component is not detrimental to patellofemoral biomechanics in posterior-referencing cruciate-retaining total knee arthroplasty

**DOI:** 10.1007/s00167-018-4900-z

**Published:** 2018-03-20

**Authors:** Marco A. Marra, Marta Strzelczak, Petra J. C. Heesterbeek, Sebastiaan A. W. van de Groes, Dennis Janssen, Bart F. J. M. Koopman, Nico Verdonschot, Ate B. Wymenga

**Affiliations:** 1 Orthopaedic Research Laboratory, Radboud Institute for Health Sciences, Radboud University Medical Center, Postbus 9101, 6500 HB Nijmegen, The Netherlands; 2 Sint Maartenskliniek Research, Postbus 9011, 6500 GM Nijmegen, The Netherlands; 3 Orthopaedic Department, Radboud University Medical Center, Postbus 9101, 6500 HB Nijmegen, The Netherlands; 4 Department of Biomechanical Engineering, University of Twente, Postbus 217, 7500 AE Enschede, The Netherlands; 5 Sint Maartenskliniek Orthopaedics, Postbus 9011, 6500 GM Nijmegen, The Netherlands

**Keywords:** Flexion, Femoral, Component, Sagittal, Alignment, Musculoskeletal, Model, CR, TKA, Biomechanics, Patellofemoral, Quadriceps, Force, Chair, Rising, Total knee arthroplasty, Total knee replacement, Posterior-referencing

## Abstract

**Purpose:**

When downsizing the femoral component to prevent mediolateral overhang, notching of the anterior femoral cortex may occur, which could be solved by flexing the femoral component. In this study, we investigated the effect of flexion of the femoral component on patellar tendon moment arm, patellofemoral forces and kinematics in posterior-referencing CR-TKA. Our hypothesis was that flexion of the femoral component increases the patellar tendon moment arm, reduces the patellofemoral forces and provides stable kinematics.

**Methods:**

A validated musculoskeletal model of CR-TKA was used. The flexion of the femoral component was increased in four steps (0°, 3°, 6°, 9°) using posterior referencing, and different alignments were analysed in combination with three implant sizes (3, 4, 5). A chair-rising trial was analysed using the model, while simultaneously estimating quadriceps muscle force, patellofemoral contact force, tibiofemoral and patellofemoral kinematics.

**Results:**

Compared to the reference case (size 4 and 0° flexion), for every 3° of increase in flexion of the femoral component the patellar tendon moment arm increased by 1% at knee extension. The peak quadriceps muscle force and patellofemoral contact force decreased by 2%, the patella shifted 0.8 mm more anteriorly and the remaining kinematics remained stable, with knee flexion. With the smaller size, the patellar tendon moment arm decreased by 6%, the quadriceps muscle force and patellofemoral contact force increased by 8 and 12%, and the patellar shifted 5 mm more posteriorly. Opposite trends were found with the bigger size.

**Conclusion:**

Flexing the femoral component with posterior referencing reduced the patellofemoral contact forces during a simulated chair-rising trial with a patient-specific musculoskeletal model of CR-TKA. There seems to be little risk when flexing and downsizing the femoral component, compared to when using a bigger size and neutral alignment. These findings provide relevant information to surgeons who wish to prevent anterior notching when downsizing the femoral component.

**Electronic supplementary material:**

The online version of this article (10.1007/s00167-018-4900-z) contains supplementary material, which is available to authorized users.

## Introduction

Implant alignment in total knee arthroplasty (TKA) is a key factor to restore natural knee kinematics and physiological loads in the tibiofemoral (TF) and patellofemoral (PF) joints, yet sagittal plane alignment of the femoral component has received relatively little attention with respect to function and outcome [13]. Previous studies recommended that the flexion of the femoral component (FFC) should be within 0°–3°, to reduce the risk of implant failure [17] and to limit the incidence of flexion contracture [19]. However, these studies addressed posterior-stabilised (PS) TKA only.

Sagittal alignment is also related to the size of the femoral component, as implants aligned in flexion have typically smaller sizes [7]. This interplay often resides in the attempt to prevent mediolateral overhang. Sometimes, the femoral component is too wide in the mediolateral dimension, which irritates the surrounding soft tissues [4]. In this situation, the surgeon typically resorts to a smaller size. However, a smaller size, in turn, increases the chance of notching of the anterior femoral cortex in non-gender specific implants. Therefore, additional flexion of the femoral component is necessary to prevent notching, when using a smaller size.

In adjusting the flexion of the femoral component, the outcome may be different depending on implant design and the surgical technique utilised. With anterior referencing, the anterior femoral cortex serves as a reference for the anterior distal femur resection, thus notching is avoided. However, this technique has the disadvantage of producing variable resection of the posterior femoral condyles with subsequent difficult balancing of the flexion space [11], and the outcome may be influenced by the type of implant chosen (single- or multi-radius design). Furthermore, because the posterior condylar offset (PCO) is not controlled, subtle increments in FFC can tighten the flexion gap substantially, as a result of over-stretching of the posterior cruciate ligament (PCL) [21]. Therefore, controlling the PCO appears essential to achieve a good TF stability. This can be achieved using posterior-referencing technique, in which the posterior femoral condyles serve as reference for the posterior resection. However, the anterior resection becomes more variable and subject to notching [11].

Flexing and downsizing the femoral component could be a solution to prevent anterior notching, alternative to a larger size. However, the effect of FFC on PF joint forces and kinematics remains largely unclear. Previous cadaver and clinical studies could not separate the effect of FFC from that of other possible confounding variables (e.g. PCO), and have shown contrasting results [5, 6, 23, 25].

The present study examines the effect of FFC and implant size on quadriceps moment arm, PF contact forces and kinematics in posterior-referenced CR-TKA, using a highly-controlled study design, in which all variables are controlled for, thus overcoming the limitations of previous cadaver studies and clinical trials. The hypothesis was that flexing and downsizing the femoral component would result in similar PF contact forces and equally stable kinematics as with neutrally-aligned upper-size implant. If this hypothesis was confirmed, then FFC could represent a viable surgical option to reconstruct the knee extensor mechanism.

## Materials and methods

For this study, a validated patient-specific musculoskeletal model was used. The creation and validation processes are described elsewhere [20]. Briefly, the model was developed using the AnyBody Modeling System (AMS, version 6, AnyBody Technology A/S, Aalborg, Denmark), it was constructed based on medical images of a patient with a telemetric CR-TKA implant, and it was validated against experimental measurements of TF contact forces and sagittal plane kinematics. In the present study, specific changes to the original model were made, which are detailed in a separate additional file [see Additional file 1]. Geometries of pre- and post-operative bones, and of the TKA implant, were obtained from an open-access dataset [12]. The femoral component was the size 4 of the Natural Knee CR-TKA system (Zimmer Biomet, Warsaw, IN, U.S.). The femoral component had a J-curved multi-radius design. The patella was resurfaced. Based on the post-operative model reconstruction in the AMS, the FFC angle was measured as the angle between the vertical axis of the femoral component and the mechanical axis of the femur. The vertical axis of the femoral component was the line perpendicular to the distal flat inner facet of the implant, and the mechanical axis of the femur was the line passing through the centre of the hip joint and the midpoint between the medial and lateral femoral epicondyles. The post-operative FFC angle was equal to 0° (neutral alignment) and represented our reference case.

**Fig. 1:**
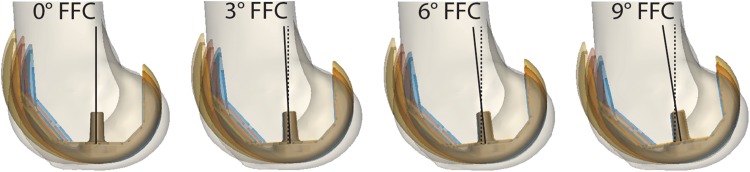
Twelve simulated post-operative cases with three different sizes and four degrees of flexion of the femoral component. Illustration of the twelve custom post-operative cases simulated in this study. From left to right four degrees of flexion of the femoral component are shown: 0°, 3°, 6°, 9°. Three sizes of the femoral component (blue: size 3, red: size 4, yellow: size 5) plus the pre-operative bone are shown in overlay for each flexion of the femoral component (FFC) angle. Note that in every case the most distal and most posterior ends of the outlines of the femoral component are made to match tangentially, to simulate a posterior referencing and to preserve the posterior condylar offset

One smaller size (size 3) and one bigger size (size 5) and three more FFC cases (+ 3°, + 6°, + 9°) were created, based on the reference model. These will be referred to as the custom post-operative cases. Geometrical models for size 3 and 5 of the femoral component were made available to us by courtesy of Zimmer Biomet (Warsaw, Indiana, U.S.). All custom cases were obtained keeping the joint space in flexion and in extension equal to that of the reference case (posterior referencing). To that aim, the femoral component geometry was translated and rotated in the sagittal plane, with the aid of the 3-D manipulation software Meshlab [8], such that its outline would always match tangentially the outline of the reference case at the most posterior and most distal ends of the implant (Fig. 1). This allowed for preservation of the post-operative PCO and did not alter the joint line in extension. Geometrical wrapping surfaces guided the path of muscles and ligament around the knee joint, and were adapted for each combination of implant size and FFC. The same size of the tibial component as of the reference case was used in all custom cases.

In addition, an intact knee case was implemented, based on pre-operative CT images of the same patient. Given the scarce visibility of menisci and cartilaginous tissues on CT images, the articular surfaces of the tibial, patellar and femoral cartilage were estimated using an offset of the bony surfaces of tibia, patella and femur, respectively. The amount of offset was made equal to the average cartilage thickness found in the literature for each respective compartment [9]. Menisci were not modelled. The anterior cruciate ligament was modelled as a spring with mechanical properties adapted from the literature [3].

The model was configured to simulate a rising-from-a-chair activity, which was recorded using standard motion capture techniques and available as part of an open-access dataset (PS_chairrise1) [12]. The trial consisted of a rising phase followed by a sitting phase for a total duration of 4.375 s. The range of knee flexion, as measured, was approximately 10°–96° and the chair-rise task was performed without the aid of the arms. Additional movie files show the musculoskeletal model in motion during a representative simulation [see Additional file 2 and 3]. The following parameters were continuously recorded as output of the simulations: patellar tendon moment arm (PTMA), patellar tendon force (PTF), quadriceps muscle force (QMF), quadriceps tendon-to-femur force (QTFF), PF contact force (PFCF), PF antero-posterior translation, the force in the PCL and medial patellofemoral ligament (MPFL) and the kinematics of the TF contact point. The PF antero-posterior translation was defined using a well-established knee joint coordinate system [14], adapted to describe PF kinematics. The femoral reference frame was built from the mechanical and transepicondylar axes of the femur, and the patellar reference frame was built based on anatomical landmarks identifying the most proximal and most distal, and the most medial and most lateral points of the patella.

A total of thirteen (three sizes and four FFC angles, plus one intact case) simulations were executed. The results of the custom post-operative cases were compared to those of the reference case (neutrally aligned, size 4). The PTMA and the PF antero-posterior translation from all post-operative cases were also compared to those obtained with the intact knee simulation. Joint forces were expressed as fractions of body weight (BW) and the ligament forces were expressed in units of newton (N).

## Results

### Patellar tendon moment arm

**Fig. 2:**
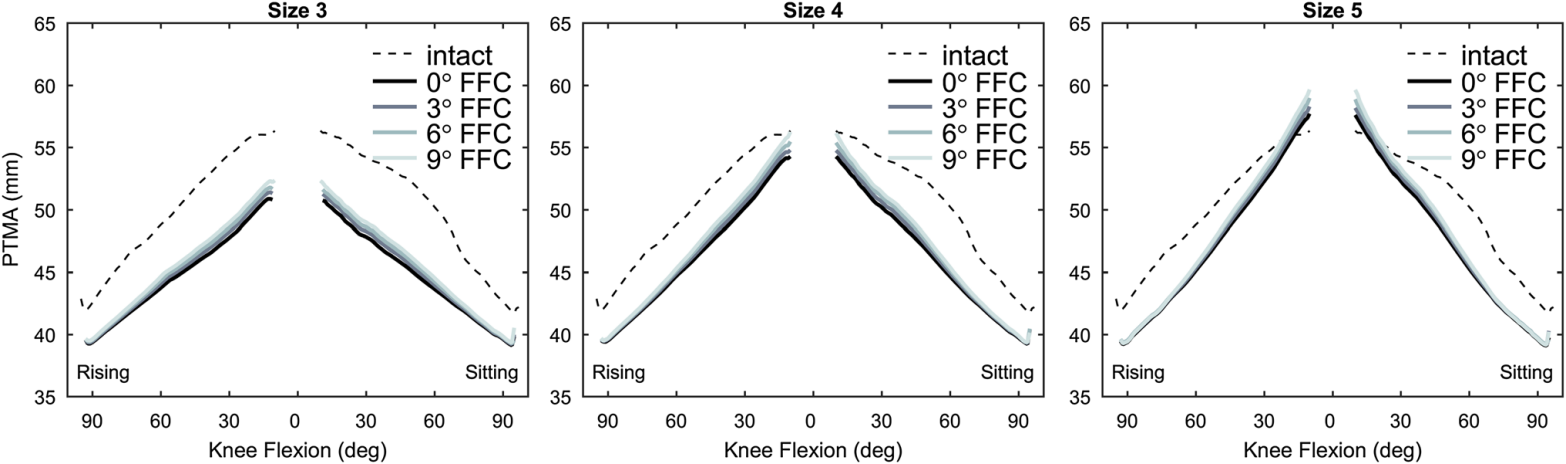
Patellar tendon moment arm. Patellar tendon moment arm (PTMA) at varying knee flexion angle during a rising-from-a-chair simulation. From left to right the results in mm for size 3, 4 and 5 are shown. Each line series correspond to a flexion of the femoral component (FFC) angle. The flexion angle in the abscissa indicates the phases of the rising and sitting motion

At knee flexion, both size and FFC had negligible effects on the PTMA. At knee extension, the PTMA increased with FFC and with a bigger size, and decreased with a smaller size, compared to the reference case (Table 1; Fig. 2). In all post-operative cases, the PTMA was about 6% smaller than in the intact case. Detailed values of PTMA for all simulated cases are provided separately (see Additional file 4).

**Table 1:** Changes in knee extensor parameters due to flexion and size of the femoral component

	+ 3° FFC	Size +	Size −
PTMA^flex^	0%	0%	0%
PTMA^ext^	+ 1%	+ 6%	− 7%
PTF	− 2%	− 5%	+ 7%
QMF	− 2%	− 7%	+ 8%
QTFF	+ 2%	+ 11%	− 15%
PFCF	− 2%	− 10%	+ 12%

Changes of patellar tendon moment arm at knee flexion (PTMA^flex^), at knee extension (PTMA^ext^), peak patellar tendon force (PTF), quadriceps muscle force (QMF), quadriceps tendon-to-femur force (QTFF), and patellofemoral contact force (PFCF) during rising-from-a-chair simulations due to varying size and flexion of the femoral component (FFC). Variations are expressed as average percentage increase (+) or decrease (-) relative to the reference case (size 4, 0° FFC) for every 3° increase of FFC (+ 3° FFC) and for a bigger size (Size +) and a smaller size (Size −)

### Forces on the knee extensor mechanism

**Fig. 3:**
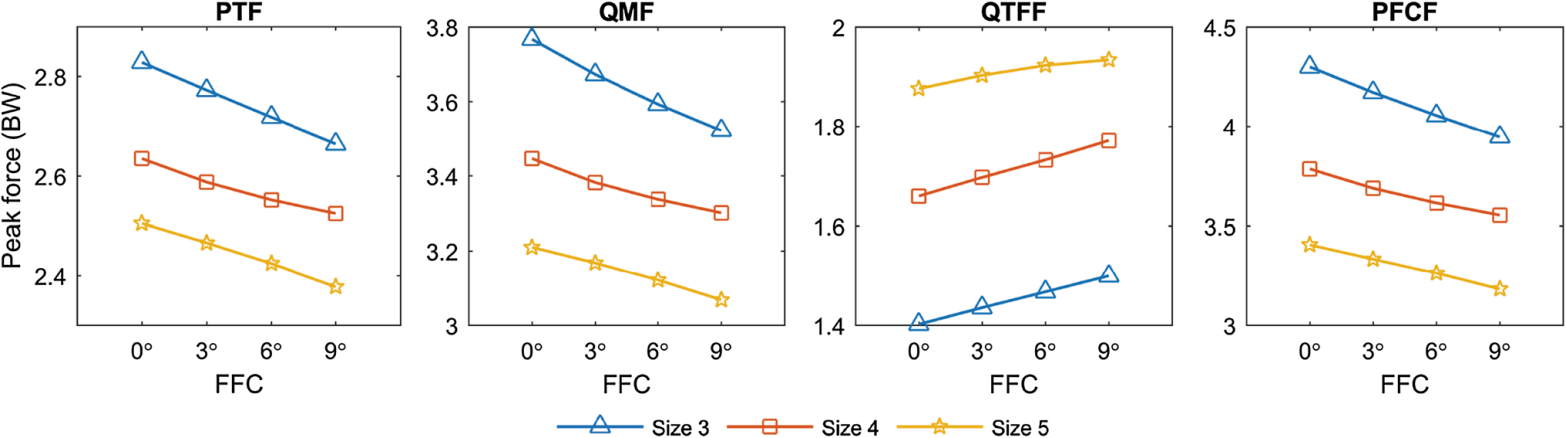
Peak forces on the knee extensor mechanism. Peak forces on the knee extensor mechanism during a rising-from-a-chair simulation. From left to right: patellar tendon force (PTF), quadriceps muscle force (QMF), quadriceps tendon-to-femur force (QTFF), and patellofemoral contact force (PFCF). Results are reported in body weights (BW)

The forces in the knee extensors mechanism peaked during the ascending phase, at a knee flexion angle of about 90 degrees. Peak values of PTF, QMF, QTFF, and PFCF for all simulated cases are depicted in Fig. 3, and their variations relative to the reference case are summarised in Table 1. Detailed peak values for all simulated cases are provided separately [see Additional file 4].

### Patellofemoral kinematics

**Fig. 4:**
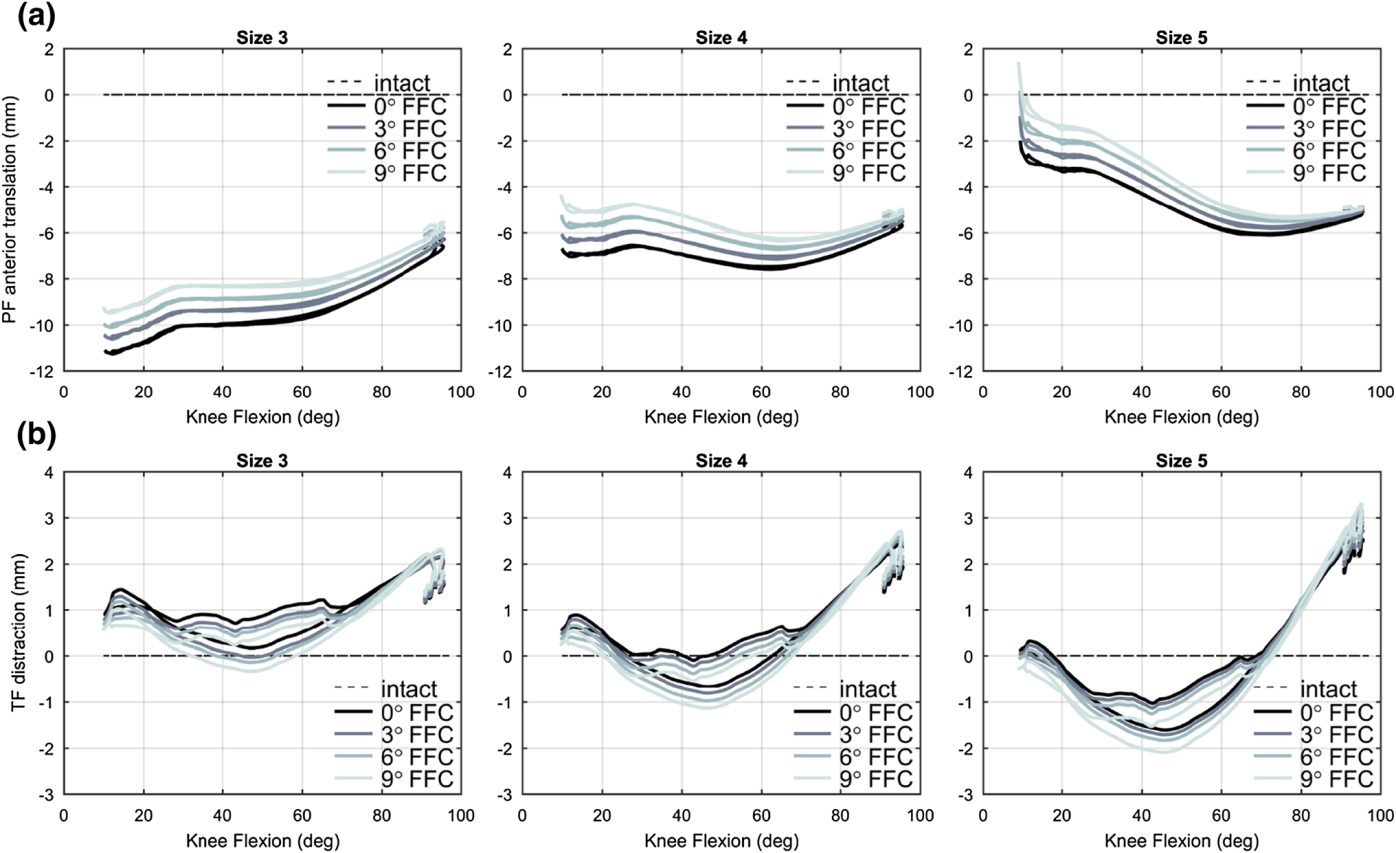
Tibiofemoral distraction and patellofemoral antero-posterior translation. Kinematics of **a** patellofemoral antero-posterior translation and **b** tibiofemoral distraction, at varying knee flexion angle during a rising-from-a-chair simulation. From left to right the results in mm for size 3, 4, and 5 are shown. Each line series correspond to a flexion of the femoral component (FFC) angle. Kinematics from the custom cases are plotted relatively to the intact case. The rising and sitting phases for each curve are overlapped

Changes in FFC and size affected the patellar antero-posterior translation (Fig. 4), and the effect was smaller with increased knee flexion. At knee extension (approximately 10° knee flexion), the patella shifted by 0.6, 0.8, and 1.1 mm more anteriorly for every 3° increase of FFC, with size 3, 4, and 5, respectively, and it shifted about 5 mm more anteriorly with a bigger size of the femoral component. Compared to the intact case, the patella was located 10.2, 5.6, and 0.3 mm more posteriorly, at knee extension, with size 3, 4, and 5, respectively.

### Ligament forces

**Fig. 5:**
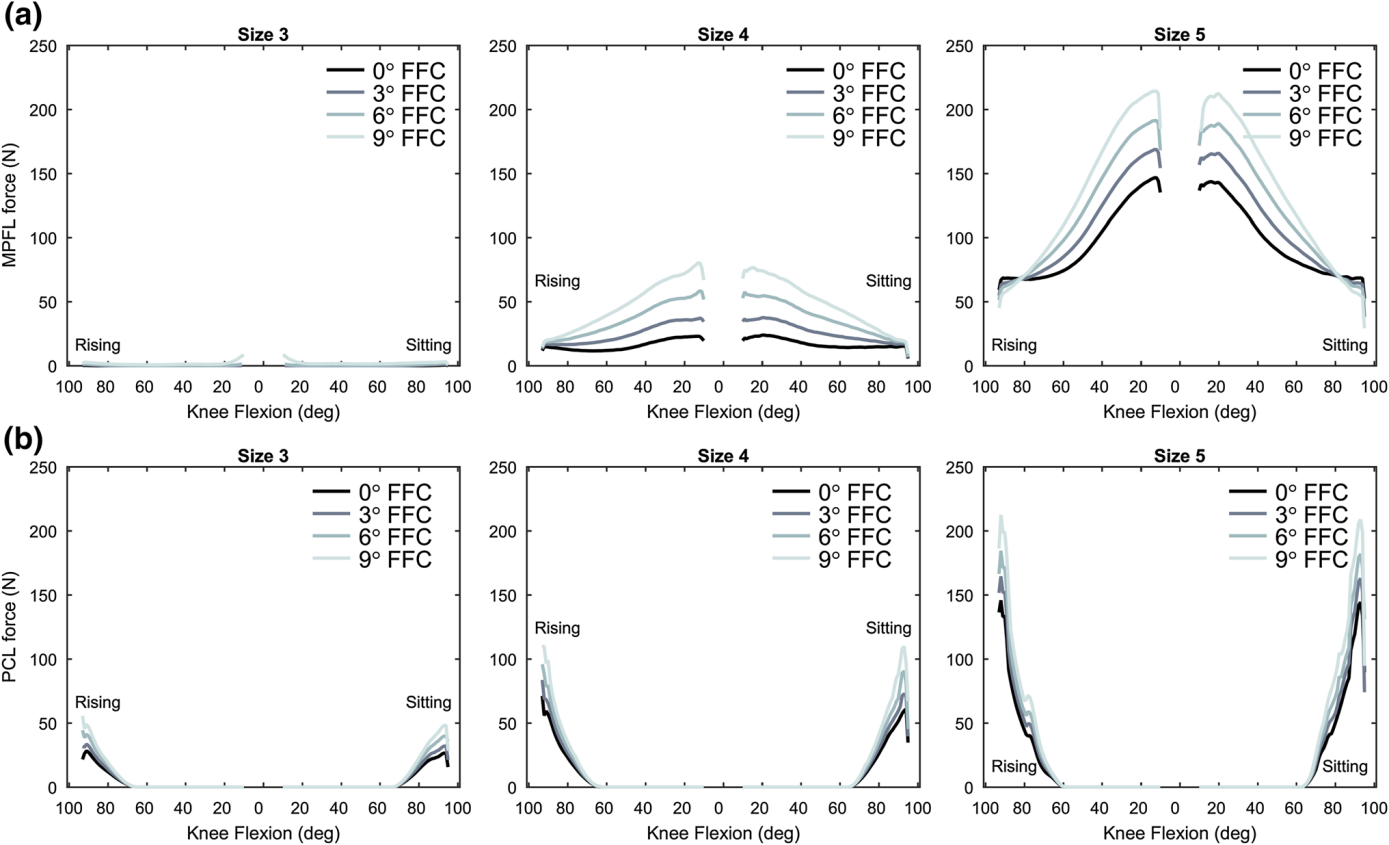
Ligament forces. Ligament force of the **a** medial patellofemoral ligament (MPFL) and **b** posterior cruciate ligament (PCL), at varying knee flexion angle during a rising-from-a-chair simulation. From left to right the results in *N* for size 3, 4, and 5 are shown. Each line series correspond to a flexion of the femoral component (FFC) angle. The flexion angle in the abscissa indicates the phases of the rising and sitting motion

The ligament forces were rather sensitive to changes in size and FFC. The MPFL force peaked at knee extension and the PCL force peaked at approximately 90° of knee flexion (Fig. 5), in the reference case. On average, the peak force in the MPFL increased by 80% for every 3° increase of FFC, especially with knee extension and mid-flexion, and increased by 314% with a bigger size. The MPFL remained slack with size 3 regardless of the FFC angle. The peak force in the PCL increased by 18%, for every 3° increase of FFC, increased by 96% with a bigger size and decreased by 56% with a smaller size.

### Kinematics of the tibiofemoral contact point

The effect of FFC on the kinematics of the TF contact point was very small. The size of the femoral component had a slightly larger effect on the kinematics (see Additional file 5). A comparison of the kinematics of the TF contact point with the intact case is also provided separately (see Additional file 6).

## Discussion

The two most important findings of this study are that flexing the femoral component: (1) while keeping the size, increases the knee extensor moment arm in extension, reduces the quadriceps and patellofemoral contact forces in flexion, and provided stable kinematics throughout the range of knee flexion and extension; (2) in combination with a smaller size, results in similar forces and kinematics as with a bigger size which is neutrally aligned. These results confirm our hypothesis and suggest that the femoral component can be downsized and flexed, to prevent both mediolateral overhang and anterior notching of the femur, and that this would result in an equally stable reconstruction of the knee extensors mechanism as with a neutrally-aligned upsized implant.

The computational approach used in this study presented some key novel aspects. It enabled the study of size and sagittal alignment of the femoral component in a single subject case, while all the other variables were unchanged, such as the PCO, the size and alignment of the tibial and patellar components, and the level of the joint line in extension. This aspect overcomes one big limitation of clinical studies, in which confounding variables are present inevitably. For instance, Antony et al. found a correlation between higher FFC and larger maximal post-operative flexion angle in CR-TKA [2], whereas Murphy et al. observed a larger maximal knee flexion angle at surgery, which did not translate in a functional benefit at 1 year post-operatively [22]. In both studies, the PCO was not controlled for, which may have acted thus as a confounding parameter.

Flexing the femoral component provided some positive effects. On the one hand, a more flexed implant increased the patellar tendon moment arm at knee extension and, to a lesser extent, in mid-flexion, which may be relevant for those activities involving large quadriceps action in the first arc of the knee range of motion. This first mechanism can be explained by the trochlear groove positioned more anteriorly and distally with more FFC. In other words, the patellofemoral joint becomes overstuffed. On the other hand, more FFC increased the QTFF in (mid-)flexion. This second mechanism redistributes some of the patellofemoral joint to the quadriceps tendon–femur compartment. A higher QTFF may result in larger stresses at the implant-bone (or implant-cement) interface, which may have an effect on implant fixation. However, these aspects were not investigated in the present study and warrant further attention. Summed together, the abovementioned effects of FFC provided a means for reducing the quadriceps and patellofemoral contact forces during dynamic and weight-bearing exercise.

A larger size of the femoral component, leaving the PCO unchanged and increasing the offset of the trochlea (posterior referencing), relative to the reference case, resulted in an even larger reduction in the quadriceps and PF forces with knee flexion from 0 to 100°, in the present study. This seems to be in contrast with the finding of Kawahara et al., who found higher PF contact forces at flexion angles of 90° and more with larger femoral components [16]. These authors, however, adopted an opposite approach: they increased the antero-posterior dimension of the femoral component by increasing the PCO and leaving the position of the anterior flange unchanged (anterior referencing). Moreover, they only evaluated PF contact forces in deeper flexion under static and non-weight-bearing conditions, and they used PS-TKA. In contrast, we estimated PF contact forces in a CR-TKA model during a dynamic and weight-bearing knee exercise, involving quadriceps muscle activity. Their findings, in essence, do not conflict with our results.

Ligament tensions here presented were in line with previous studies on ligament length changes in TKA [1, 15, 18]. With a bigger size, the both PCL and MPFL forces increased substantially, and much more than observed after variations in FFC alone. Higher tension in the MPFL resulted from an oversized femoral component (mediolateral overhang), and this may be detrimental to the results of TKA [4]. For this reason, over-sizing the femoral component is generally discouraged. Larger PCL forces with a bigger size of the femoral component were in agreement with findings of previous studies [10], and could be explained both by a larger TF distraction and a larger posterior tibial translation with knee flexion. In contrast, a smaller femoral component slackened the MPFL nearly entirely, due to a posterior patellar translation (understaffing) and a smaller mediolateral size of the femoral component, and the PCL force was halved, compared to the reference size. This scenario is also discouraged, as slackening of the MPFL may increase the risk of patellar instability (although no aberrant PF kinematics were observed in this study) [24] and slackening of the PCL may destabilise the knee in flexion. Flexing the femoral component could partially restore the tension in these ligaments.

The post-operative PTMA in (mid-)flexion was consistently smaller than in the intact case, which may indicate a failed reconstruction of the PTMA for other reasons. At knee extension, similar PTMA was obtained in the intact case, with size 5, and with size 4 with additional FFC. Therefore, increasing the FFC may also increase the PTMA in extension. Implant size had the largest influence on patellar antero-posterior translation. Post-operatively, the patella was consistently less anterior than in the intact case, throughout the range of flexion–extension. In extension and mid-flexion, additional FFC could partially restore the antero-posterior translation.

**Fig. 6:**
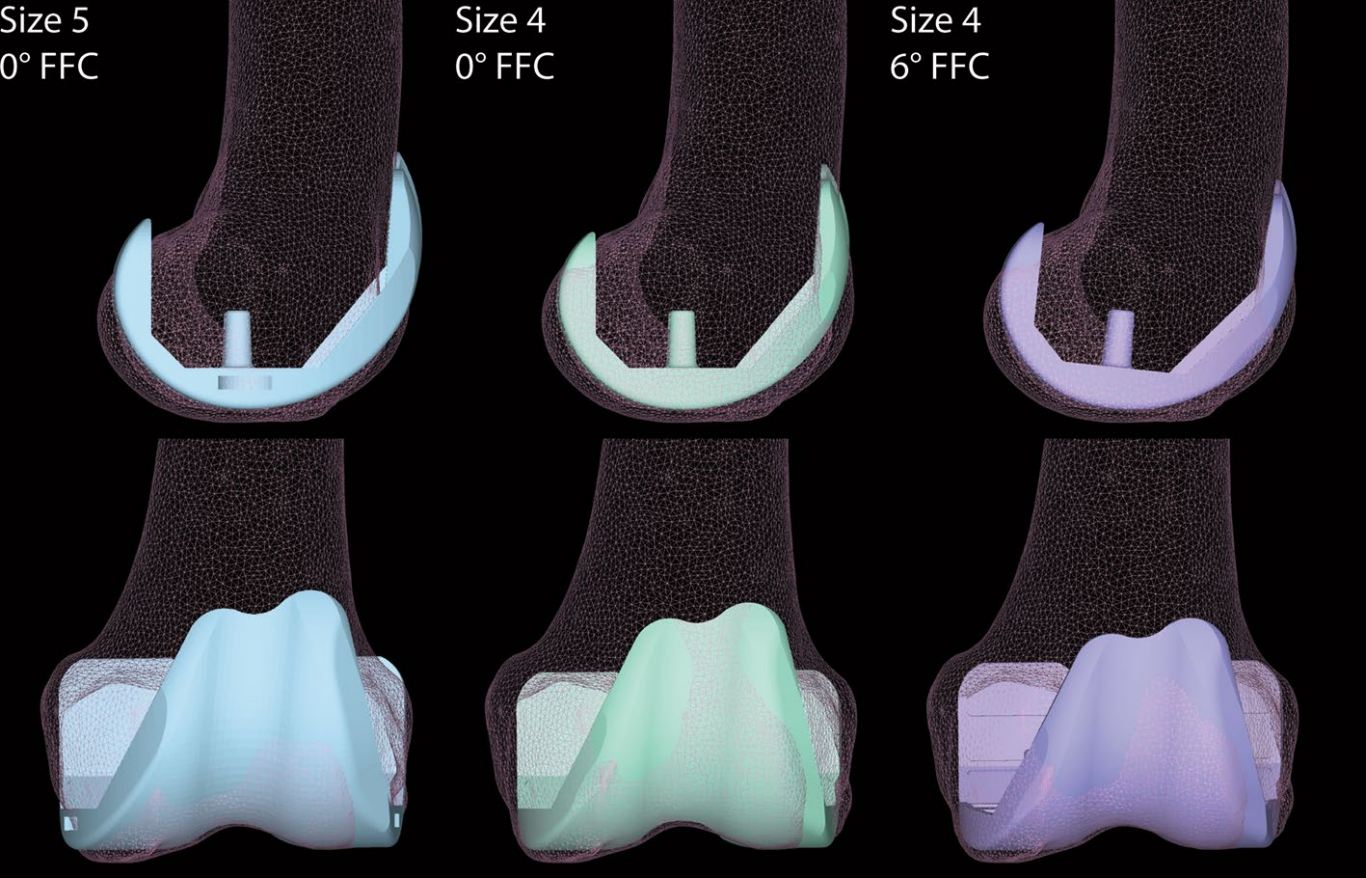
Illustrative case for the alignment in flexion of a downsized femoral component. Illustrative case for the alignment in flexion of a downsized femoral component with preservation of the posterior condylar offset (PCO). Size 5 with 0° FFC fits the antero-posterior dimension of the femur, however, mediolateral overhang is observed, which is detrimental. Downsizing the femoral component (Size 4, 0° FFC) reduces the mediolateral overhang, but creates anterior notching of the femoral cortex, if the PCO is preserved. Flexing the smaller component by a few degrees in the sagittal plane (Size 4, 6° FFC) may concomitantly preserve the PCO, while limiting mediolateral overhang and preventing anterior notching

From a purely anatomical point of view, and if we consider only the femoral antero-posterior dimension, the size 5 of the femoral implant would likely provide the best fit (Fig. 6). However, such a choice could be less favourable concerning mediolateral overhang, as it could consequently cause an irritation of the soft tissues. Virtually, an equally good antero-posterior fit as with size 5 could be achieved using a smaller femoral component (size 4) which is flexed by about 6°. Despite the downsizing, flexing the femoral component while preserving the PCO would also ensure a proper reconstruction of the flexion space, without concerns of anterior notching of the femoral cortex.

In light of these findings, flexing and downsizing the femoral component seem to provide similar biomechanical results as using a bigger size with neutral alignment, but without the problem of mediolateral overhang and anterior notching. Moreover, flexing the femoral component does not appear detrimental to TF and PF kinematics. Therefore, surgeons may consider flexing the femoral component as an option to limit anterior femoral notching in downsized implant. Surgeons should also be aware that downsizing the femoral component might decrease the tension in the PCL and MPFL, and flexing the femoral component may partially restore this tension, as shown in this study.

The present study elucidates biomechanical aspects of sagittal alignment and size of the femoral component in CR-TKA with posterior referencing. Caution should be used when generalising the present findings to other implant types (e.g. PS-TKA), designs (e.g. single-radius) and surgical techniques (e.g. anterior referencing), and cases of large anatomical deformity, as these were not investigated. Furthermore, given our choice to preserve the PCO with posterior referencing, some of the simulated cases (e.g. size 3 with 0° and 3° FFC and size 5 with 6° and 9° FFC) are not plausible in practice. These hypothetical cases were included as well, to provide a more comprehensive overview of the parameters investigated. The use of a computer model to simulate the effect of size and alignment involved many assumptions and simplifications. The musculoskeletal model was based on only one patient and implant design, which minimised possible confounding variables. Future research should assess the influence of anatomical variability and validate these findings in a clinical setting; this study provides clues as to which parameters could be included.

## Conclusion

Flexing the femoral component increases the knee extensors moment arm and reduces the quadriceps and patellofemoral contact forces in posterior-referencing CR-TKA. There seems to be little risk associated with flexing the femoral component in a downsized implant, which could have advantages in terms of preventing mediolateral overhang and anterior notching, and would result in similar patellofemoral forces and kinematics as in a neutrally-positioned upsized component.

## Electronic supplementary material

Below is the link to the electronic supplementary material.

Supplementary material 1 (DOCX 23 KB)

Supplementary material 2 (WMV 8142 KB)

Supplementary material 3 (WMV 11416 KB)

Supplementary material 4 (DOCX 20 KB)

Supplementary material 5 (TIF 4066 KB)

Supplementary material 6 (TIF 190 KB)
