# Supplementary material for: Flexing and downsizing the femoral component is not detrimental to patellofemoral biomechanics in posterior-referencing cruciate-retaining total knee arthroplasty
Source: Knee Surg Sports Traumatol Arthrosc. 2018 Mar 20;26(11):3377–85. doi: 10.1007/s00167-018-4900-z (PMC6208942; doi:10.1007/s00167-018-4900-z)
Supplement: Supplementary file 1 — Supplementary material 1 (DOCX 23 KB) [file 167_2018_4900_MOESM1_ESM.docx]

## Details of the musculoskeletal model

The model used in this study was built upon a previously validated model of cruciate-retaining total knee arthroplasty model [4]. This model was based on the Twente Lower Extremity Model 2.0 [2], and included: head, trunk, two arms and two legs, connected by idealised joints. The bones of the lower extremities were non-linearly scaled to match those of the patient, using morphing techniques. Three-dimensional models of the CR-TKA implant were incorporated into the patient’s left knee. Time histories of reflective skin marker trajectories were used as input to derive the kinematics of the idealised joint degrees of freedom, using a motion optimization algorithm [2]. Subsequently, the tibiofemoral and patellofemoral joint constraints were removed, and ligaments and articular surface contacts were introduced to provide elastic stiffness to the unconstrained joints. Experimental ground reaction forces and optimized joint kinematics were input to a model equipped with 166 Hill-type muscle-tendon elements. Muscle and ligament forces, articular contact forces and secondary knee kinematics were solved simultaneously using force-dependent kinematics [5]. Some variations were introduced with respect to our previously published model: the patellar ligament (PL) was modelled as three non-linear elastic springs with large stiffness, in place of a rigid rod; the lateral patellofemoral ligament bundles were removed to save computation time, since they remained slack throughout a series of trial simulations; the path of the muscle *vastus medialis* was further wrapped around an ellipsoidal wrapping object, to account for the obliquity of the fascicles at its patellar insertion [1, 3].

# References

1. Bennett WF, Doherty N, Hallisey MJ, Fulkerson JP (1993) Insertion orientation of terminal vastus lateralis obliquus and vastus medialis obliquus muscle fibers in human knees. Clin Anat 6:129–134

2. Carbone V, Fluit R, Pellikaan P, van der Krogt MM, Janssen D, Damsgaard M, Vigneron L, Feilkas T, Koopman HFJM, Verdonschot N (2015) TLEM 2.0 – A comprehensive musculoskeletal geometry dataset for subject-specific modeling of lower extremity. J Biomech Elsevier 48:734–741

3. Engelina S, Antonios T, Robertson CJ, Killingback A, Adds PJ (2014) Ultrasound investigation of vastus medialis oblique muscle architecture: An in vivo study. Clin Anat 27:1076–1084

4. Marra MA, Vanheule V, Fluit R, Koopman BHFJM, Rasmussen J, Verdonschot N, Andersen MS (2015) A subject-specific musculoskeletal modeling framework to predict in vivo mechanics of total knee arthroplasty. J Biomech Eng 137:20904

5. Skipper Andersen M, de Zee M, Damsgaard M, Nolte D, Rasmussen J (2017) Introduction to Force-Dependent Kinematics: Theory and Application to Mandible Modeling. J Biomech Eng 139:91001
