# Supplementary material for: Flexing and downsizing the femoral component is not detrimental to patellofemoral biomechanics in posterior-referencing cruciate-retaining total knee arthroplasty
Source: Knee Surg Sports Traumatol Arthrosc. 2018 Mar 20;26(11):3377–85. doi: 10.1007/s00167-018-4900-z (PMC6208942; doi:10.1007/s00167-018-4900-z)
Supplement: Supplementary file 4 — Supplementary material 4 (DOCX 20 KB) [file 167_2018_4900_MOESM4_ESM.docx]

**Raw results from all simulations.**

**Patellar tendon moment arm in flexion (PTMA^flex^) during chair-rising**

|  | | Size | | |
| --- | --- | --- | --- | --- |
|  |  | 3 | 4 | 5 |
| FFC | 0° | 39.1 | 39.2 | 39.1 |
|  | +3° | 39.2 | 39.3 | 39.1 |
|  | +6° | 39.3 | 39.3 | 39.2 |
|  | +9° | 39.3 | 39.4 | 39.1 |

Patellar tendon moment arm at knee flexion (PTMA^flex^) during rising-from-a-chair simulations with three different sizes and four different femoral component flexion angles (FFC). Values are in mm.

**Patellar tendon moment arm in extension (PTMA^ext^) during chair-rising**

|  | | Size | | |
| --- | --- | --- | --- | --- |
|  |  | 3 | 4 | 5 |
| FFC | 0° | 50.9 | 54.3 | 57.7 |
|  | +3° | 51.4 | 54.8 | 58.3 |
|  | +6° | 51.8 | 55.5 | 59.0 |
|  | +9° | 52.3 | 56.2 | 59.6 |

Patellar tendon moment arm at knee flexion (PTMA^ext^) during rising-from-a-chair simulations with three different sizes and four different femoral component flexion angles (FFC). Values are in mm.

**Patellar tendon force (PTF) during chair-rising**

|  | | Size | | |
| --- | --- | --- | --- | --- |
|  |  | 3 | 4 | 5 |
| FFC | 0° | 2.83 | 2.63 | 2.50 |
|  | +3° | 2.77 | 2.59 | 2.47 |
|  | +6° | 2.72 | 2.55 | 2.42 |
|  | +9° | 2.66 | 2.52 | 2.38 |

Peak patellar tendon force (PTF) during rising-from-a-chair simulations with three different sizes and four different femoral component flexion angles (FFC). Values are expressed as fractions of body weight (BW).

**Quadriceps muscle force (QMF) during chair-rising**

|  | | Size | | |
| --- | --- | --- | --- | --- |
|  |  | 3 | 4 | 5 |
| FFC | 0° | 3.77 | 3.45 | 3.21 |
|  | +3° | 3.67 | 3.38 | 3.17 |
|  | +6° | 3.59 | 3.34 | 3.12 |
|  | +9° | 3.52 | 3.30 | 3.07 |

Peak quadriceps muscle force (QMF) during rising-from-a-chair simulations with three different sizes and four different femoral component flexion angles (FFC). Values are expressed as fractions of body weight (BW).

**Quadriceps tendon-to-femur force (QTFF) during chair-rising**

|  | | Size | | |
| --- | --- | --- | --- | --- |
|  |  | 3 | 4 | 5 |
| FFC | 0° | 1.40 | 1.66 | 1.88 |
|  | +3° | 1.44 | 1.70 | 1.90 |
|  | +6° | 1.47 | 1.73 | 1.92 |
|  | +9° | 1.50 | 1.77 | 1.93 |

Peak quadriceps tendon-to-femur force (QTFF) during rising-from-a-chair simulations with three different sizes and four different femoral component flexion angles (FFC). Values are expressed as fractions of body weight (BW).

**Patellofemoral contact force (PFCF) during chair-rising**

|  | | Size | | |
| --- | --- | --- | --- | --- |
|  |  | 3 | 4 | 5 |
| FFC | 0° | 4.30 | 3.78 | 3.41 |
|  | +3° | 4.17 | 3.69 | 3.33 |
|  | +6° | 4.06 | 3.61 | 3.26 |
|  | +9° | 3.95 | 3.55 | 3.18 |

Peak patellofemoral contact force (PFCF) during rising-from-a-chair simulations with three different sizes and four different femoral component flexion angles (FFC). Values are expressed as fractions of body weight (BW).
