# Supplementary figures and images for: Flexing and downsizing the femoral component is not detrimental to patellofemoral biomechanics in posterior-referencing cruciate-retaining total knee arthroplasty
Source: Knee Surg Sports Traumatol Arthrosc. 2018 Mar 20;26(11):3377–85. doi: 10.1007/s00167-018-4900-z (PMC6208942; doi:10.1007/s00167-018-4900-z)

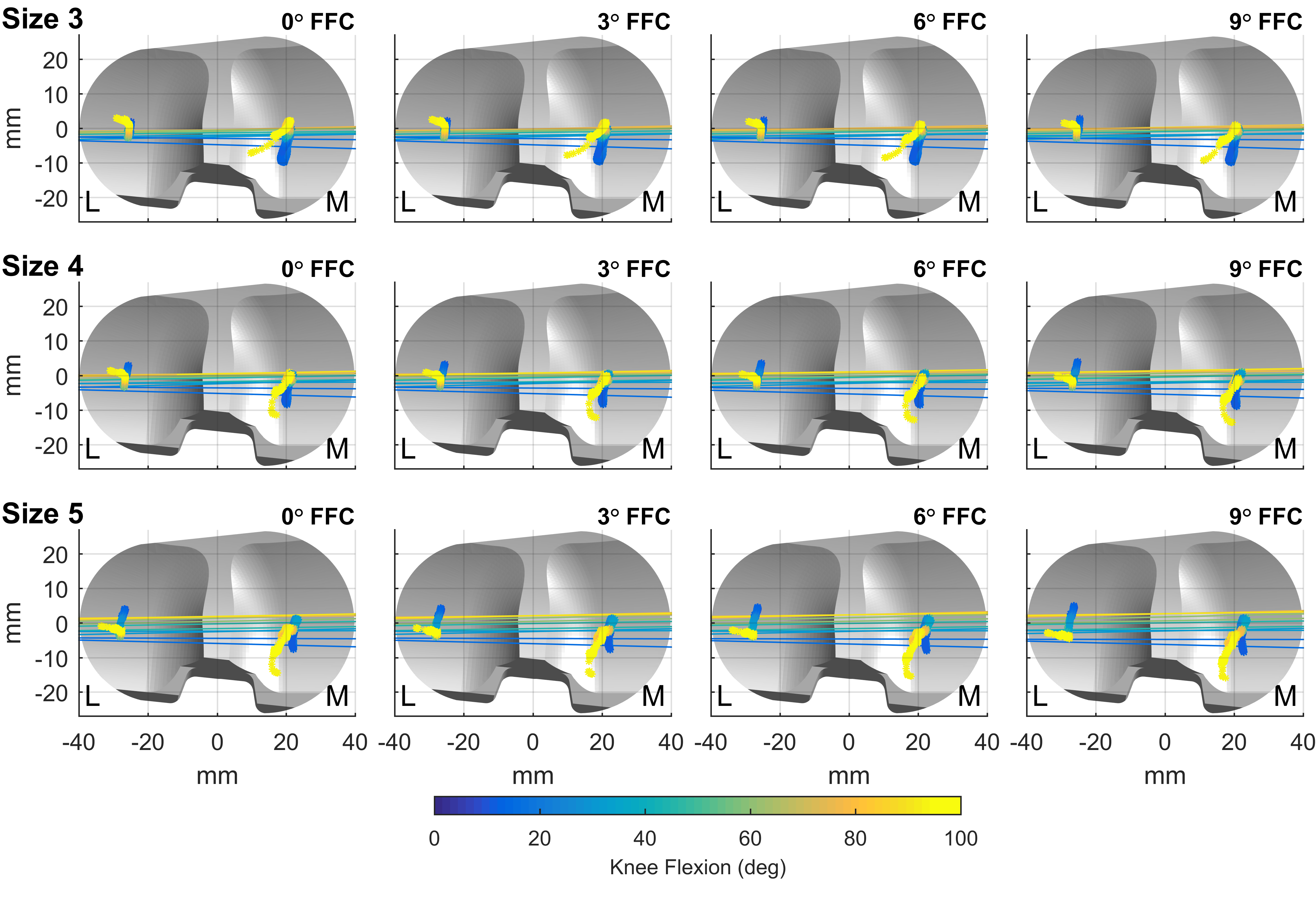

Supplement: Supplementary file 5 — Supplementary material 5 (TIF 4066 KB) [file 167_2018_4900_MOESM5_ESM.tif]

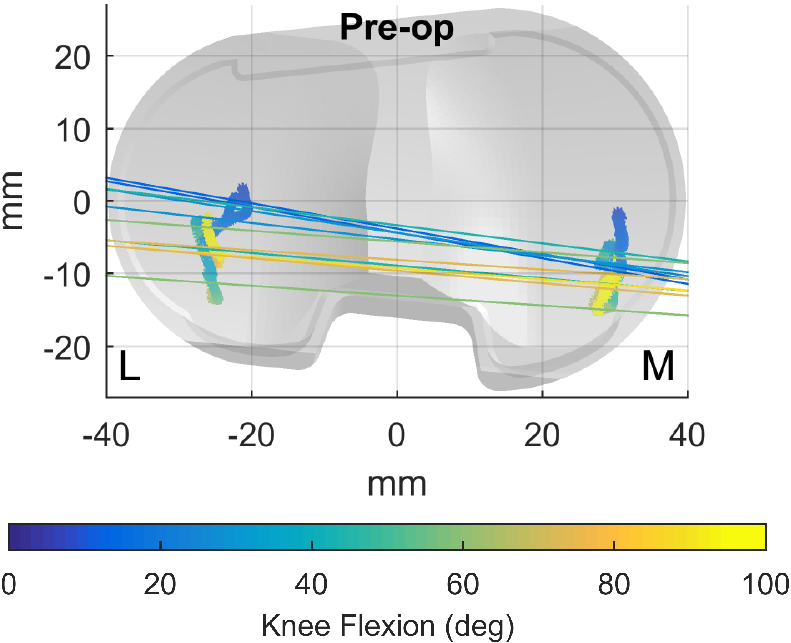

Supplement: Supplementary file 6 — Supplementary material 6 (TIF 190 KB) [file 167_2018_4900_MOESM6_ESM.tif]
